# Evolutionary Imprints on the Foliar Elementome: Biogeochemical Niche Differentiation Among *Fagus* Species

**DOI:** 10.1002/ece3.74196

**Published:** 2026-08-26

**Authors:** Daisy Cárate Tandalla, Hermann F. Jungkunst, Janice E. Hudson, Delphis F. Levia, Junko Nagakura, Josep Peñuelas, Vanessa M. S. Vetter, Jordi Sardans

**Affiliations:** ^1^ Department of Natural and Environmental Sciences RPTU Kaiserslautern‐Landau Landau Germany; ^2^ Department of Geography & Spatial Sciences University of Delaware Newark Delaware USA; ^3^ Forestry and Forest Products Research Institute Tsukuba Japan; ^4^ CSIC, Global Ecology Unit CREAF‐CSIC‐UAB Bellaterra Spain; ^5^ CREAF Cerdanyola del Vallès Spain

**Keywords:** beech, biogeochemical niche, competitive ability, Europe, forests, Japan, North America

## Abstract

Although phylogenetically related plant species often differ in competitive ability, the underlying causes remain poorly understood. The present study characterizes the biogeochemical niches (BN) of *Fagus* species to clarify interspecific variation in elemental acquisition, life history strategies, and competitive abilities. Quantifying the biogeochemical niche (BN) provides a mechanistic framework to address the shortfall in our understanding of how evolutionary history shapes trait–environment relationships and governs interspecific competition. We examined *Fagus grandifolia* Ehrh. from North America, *Fagus sylvatica* L. from Europe, and *Fagus crenata* Blume. from Asia. Foliar N, P, K, Ca, and Mg concentrations and nutrient ratios data from 328 sites found in published data were analyzed in relation to climate data. Multivariate analyses identify distinct foliar chemical signatures for each species. Phosphorus (P) and calcium (Ca) significantly differentiated *F. sylvatica* from its congeners (*p* < 0.001), while *F. crenata* exhibited the highest concentrations of K and Mg (*p* < 0.001). Squared Mahalanobis distances confirmed a clear elementome (the chemical‐atomic stoichiometrical composition) separation, with *F. sylvatica* and *F. crenata* representing the most divergent biogeochemical niches. Discriminant analysis (FDA) correctly classified > 85% of samples based on species foliar atomic composition identity, with phosphorus (P) and calcium (Ca) being the primary drivers of differentiation. Our results reveal that these congeners occupy non‐overlapping biogeochemical niches, suggesting that their respective elementomes are deeply conserved traits shaped by ancestral climate and edaphic conditions. Functional profiling reveals three distinct syndromes: *F. grandifolia* adopts a conservative, resource‐use‐efficient strategy; *F. sylvatica* exhibits a stoichiometrically flexible, acquisitive strategy consistent with its rapid post‐glacial expansion; and *F. crenata* displays an intermediate, plastic strategy. These findings indicate that competitive dominance and forest resilience in *Fagus* are underpinned by species‐specific elementomes. Consequently, climate‐driven shifts in species composition will fundamentally restructure ecosystem‐level nutrient cycling and stoichiometry.

## Graphical abstract

This study compared the biogeochemical niches of three Fagus species ( *F. grandifolia* , *F. sylvatica* , and *F. crenata* ) using foliar nutrient data from 328 sites, revealing distinct elemental compositions and unique nutrient acquisition strategies. Phosphorus and calcium primarily distinguished *F. sylvatica* , whereas *F. crenata* exhibited the highest concentrations of potassium and magnesium. These findings indicate that species‐specific elementomes represent evolutionarily conserved traits that influence functional strategies, forest resilience, and ecosystem nutrient cycling in the context of future climate change.

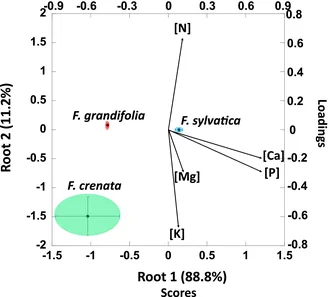

## 1 Introduction

Ecosystems across the Northern Hemisphere host major evolutionary lineages with the ability to extend across multiple ecoregions, ranging from temperate to subtropical zones. Among these, the Fagaceae family, including beeches and oaks trees, is particularly noteworthy. Within Fagaceae, *Fagus* (beech) is a key genus representative of the broadleaved deciduous tree species worldwide. Three prominent *Fagus* species: *Fagus sylvatica* L. (European beech) in Europe, *Fagus grandifolia* Ehrh. (American beech) in North America, and *Fagus crenata* Blume (Japanese beech) in Japan, share similar morphological traits (Table 1) and are, at different levels, dominant in temperate forest ecosystems. Each species, within its biogeographic ranges, constitutes a major shade‐tolerant species, co‐occurring in different species assemblages with other trees (Stephanson and Coe 2017; Hara 2023). Although the three species occupy similar bioclimatic regions, soil types (Table 1), and vegetation types as defined by the Holdridge Life Zones system (Holdridge 1947), their relative dominance differs across their respective zones. The relative competitive success of any of the three *Fagus* species *vis‐à‐vis* the other species within their respective biogeographical ranges may be obscured by their similar morphology, phenology, reproductive strategies and ecological distribution and roles as well as their ecological functional strategies inherited by evolution. To disentangle these complexities and shed light on the biogeographic distribution and functional evolutionary traits of these *Fagus* species, we turn to disentangle the differences in biogeochemical niche (BN) among these species (Peñuelas et al. 2019).

**TABLE 1:** Species and environmental characteristics of the three *Fagus* species in the Northern Hemisphere.

	*F. grandifolia* (North American beech)	*F. sylvatica* (European beech)	*F. crenata* (Japanese beech)
Tree size and shape	Height to 30+ m height, 3.5 m dbh Rounded crown. Pale gray, smooth bark	Height up to 40 m height, 1–1.3 m dbh (up to 7 m or more) Rounded or oval crown. Pale gray bark	Height to 30 m height, 1 m dbh Rounded crown. Grayish bark
Leaf morphology and Phenology	Leaves 6–12 × 2.5–7.5 cm; dark green above, pale green below; *ovate to narrowly‐ovate or obovate*; apex sub‐acuminate, base cuneate, margins sparsely but distinctively toothed; lateral veins in 9–14 pairs, petiole 4–12 mm, often hairy. Cupule brown to reddish‐brown, 1.5–2(−2.5) cm, with long recurved awns; peduncles 16–22 mm. Flowering April to June; fruiting September to October (North America) (Grimshaw and Christian 2021)	Leaves (3–)4–10 cm × 2.5–4 cm; bright green above, pale green below; *oval‐elliptic*, apex acute, base rounded, margins ciliate or remotely and very finely toothed (distinctly lobed in some cultivars); veins 5–8 pairs; petiole 0.3–1 cm. Cupule c. 2.5 cm, scales are of one kind, needle‐like. Perianth of male flowers divided almost to the base; peduncles densely pubescent, 2.5 cm. (Christian 2019)	Leaves 5–8 × 3–5 cm; mid‐green above, pale green below; *ovate to rhombic‐ovate*, broadest below the middle, apex short‐acuminate, base cuneate to rounded or shallowly cordate; margins undulate, with small or no teeth, veins 7–11 pairs; petiole 3–10 mm, slightly pubescent. Cupule c. 15 mm, with dense, linear, rather fleshy bristles 3–7 mm long, especially large and sometimes spathulate near the base; peduncles 5–15 mm, pubescent. Autumn turns rich yellow brown (Grimshaw and Christian 2021)
Reproduction	Sexual and clonal	Sexual and clonal	Sexual and clonal (Worth et al. 2021)
Ecology and main distribution	Deciduous/mixed forests, especially on cooler slopes, 0–2000 m asl, often with other temperate species. Canada (Nova Scotia, New Brunswick, southern Quebec, southeast Ontario), Eastern United States (Maine to Georgia, northern Wisconsin to eastern Texas) and Eastern Mexico (Nuevo Leon to Hidalgo, Puebla and Veracruz)	Forests on well‐drained soil, especially in hills and mountains. Not naturally at home on poorly drained soils, or in low‐lying plains, etc., but extensively planted everywhere mainly as monospecific forests. Europe (Denmark to Calabria and the mountains of northeastern Sicily, and Spain from the Catalonian Pyrenees westward as far as Turkey and parts of Ukraine) and the United Kingdom (Southern England)	Temperate mixed forests from 300 to 1600 m asl. Japan (northeast on the Japan Seaside and southwest on the Pacific Ocean side) (Sugimoto and Ishida 2023)
Soil	Primarily udic alfisols, inceptisols, and ultisols^a^	Well drained soils; alfisols, inceptisols, ultisols^a^	Alfisols, inceptisols, ultisols^a^
Holdridge Life Zones (Koppen climate^b^)	Subtropical moist forest (Cfa), Cool temperate forest (Cfb), Cool temperate wet forest or Subtropical moist (Dfa), Cool temperate wet to Boreal moist (Dfb)	Subtropical moist forest (Cfa), Cool temperate forest (Cfb), Cool temperate wet forest or Subtropical moist (Dfa), Cool temperate wet to Boreal moist (Dfb)	Subtropical moist forest (Cfa), Cool temperate forest (Cfb), Cool temperate wet forest or Subtropical moist (Dfa), Cool temperate wet to Boreal moist (Dfb)

^a^ (Soil Survey Staff. USDA Natural Resources Conservation Service‐NRCS 2023).

^b^ Cfa = humid subtropical climate; Cfb = temperate oceanic climate or subtropical highland climate; Dfa = hot‐summer humid continental climate; Dfb = warm‐summer humid continental climate.

The BN hypothesis builds on the fact that the main determinant of a species' elemental composition in its foliar biomass lies mostly in its long‐term evolutionary processes, which shape each species, leading to distinct functional and morphological structures that drive the differential use of macro‐ and micronutrients. Thus, each species should have an optimal equilibrium of bioelements composition (i.e., micro‐ and macronutrients necessary for its morphological and physiological performance) involved in its individual functional and structural adaptation, defined as the “elementome” (Peñuelas et al. 2019). The hypothesis suggests that, at equilibrium, coexisting species tend to develop distinct elementomes to minimize competitive pressure. These differences in elemental composition can confer functional advantages or disadvantages depending on the environment. Yuan et al. (2024) observed this functional differentiation in a regional study in China. Slow‐growing woody species, often competitors or stress tolerators, exhibited more conservative stoichiometric traits (e.g., higher leaf Ca and Mn), which may enhance structural stability, stress resistance, and resource retention under stable or nutrient‐limited conditions. In contrast, fast‐growing herbaceous species displayed flexible stoichiometric traits (e.g., higher leaf N, K, Zn, Fe), supporting rapid nutrient uptake, high metabolic rates, and faster growth traits advantageous in resource‐rich or fluctuating environments (ruderal specialists). These results suggested that trade‐offs reflect a continuum between conservative and acquisitive strategies influencing how species respond to competition and environmental variability. Thus, BN distances among species are shaped not only by taxonomic differences but also by sympatry (coexistence), where species‐specific degrees of stoichiometric homeostasis and plasticity matter (Peñuelas et al. 2008, 2019). Although each plant species possesses a distinct foliar elemental composition adapted to its specific traits, it also has an inherit capacity to adjust to prevailing climatic conditions and competitive environments. This adaptability enables species to respond to environmental changes or the presence of neighboring species (Peñuelas et al. 2008, 2010, 2019; Sardans et al. 2015, 2021). Consequently, co‐occurring species tend to reduce direct competition for identical soil resources by occupying distinct “niche spaces,” thereby optimizing species‐specific functions and the exploitation of soil resources.

The first paradigm of the BN hypothesis suggests that each species is a unique genetic pool shaped by long‐term evolution. Each species, therefore, develops its own specific morphology and function, from gene expression to physiology. Major biological processes, such as growth, secondary metabolism, reproduction and element storage, occur at distinct rates in different species. As a result, species allocate elements differently among their tissues and organs. Typically, each species maintains a unique elemental composition and stoichiometry (the homeostatic component of BN). However, changing environments require individuals to adapt to their function and structure during life (the plasticity component of BN). When testing the BN of different species, it is essential to consider how much variance in elemental composition is attributable to legacy (heritability) (Peñuelas et al. 2019; Sardans et al. 2021, 2023; Hu et al. 2025).

Recent studies (Hu et al. 2025; Wang et al. 2025) applied this approach to differentiate the legacy resulting from long‐term evolutionary separation among species or genotypes (phylogenetic effects) from those unrelated to phylogeny, such as recent convergent and divergent evolutionary processes (Sardans et al. 2021). For example, some of the phylogenetic lineages from distant clades encountered similar environmental conditions, which likely drove parallel selection for traits that influence elemental concentrations. This pattern can, therefore, lead to convergence toward more similar elementomes than expected based on phylogenetic distance (Gillman et al. 2010; Puurtinen et al. 2016). Conversely, recent divergent evolution may occur among closely related species adapted to distinct and divergent environmental conditions within their respective distribution areas (Sardans et al. 2021, 2023). While the phylogenetic history of *Fagus* is well studied, functional trait‐based methods such as elementome analyses are still largely unexplored for understanding the mechanisms behind the group's wide distribution and ecological adaptation. Here, we use elemental leaf composition as a proxy to characterize the BN of the three main *Fagus* species: *F. grandifolia* (North America, United States), *F. sylvatica* (Europe, several countries), and *F. crenata* (Asia, Japan) from three different continents.

The primary claim of this study is that differences in elementomes among the three chosen *Fagus* species reflect divergent evolutionary trajectories shaped by both historical habitat separation and differential human impact. Specifically, we compare the elementomes of the three beech species across their natural biogeographic ranges to identify contrasting functional strategies and characterize their biogeochemical niches.

Based on current and historical distribution ranges, we expect that *F. sylvatica* will exhibit a broader and more acquisitive biogeochemical niche due to its post‐glacial expansion history and adaptation to a wider range of management‐induced disturbances, while *F. grandifolia* and *F. crenata* are anticipated to occupy narrower biogeochemical niches. Accordingly, we expect that *F. sylvatica* exhibits traits associated with rapid growth, including elevated phosphorus (P) concentration and low N:P ratios, as outlined by the growth rate hypothesis (Elser et al. 2000). The present analysis will provide insights into the functional strategies associated with their contrasting ecological dominance across comparable bioclimatic regions.

## 2 Methods

### 2.1 Species Studied

According to the latest group phylogenies, the genus *Fagus* includes eleven native species distributed in the three continents of the northern hemisphere (Jiang et al. 2022). The genus evolved gradually and spread bidirectionally. From the original distribution in South Asia (oldest species: *Fagus engleriana, F. japonica, F. multinervis*), it reached North America around 43.6 Myr as *F. grandifolia* , Central Europe around 40.2 Myr as *F. sylvatica* and *F. orientalis* in the middle Eocene, to arrive later to Southeast Asia, around 23.2 Myr as *Fagus hayata, F. crenata, F. longipetiolata*, and *F. lucida* , in the early Miocene according to Renner et al. (2016). Another comprehensive phylogeny published by Jiang et al. (2022) suggests similar ancestral reconstruction of the genus but pointed out a relatively earlier time of species divergence. We selected three species that depict *Fagus*' evolutionary history and extended representation in the Northern Hemisphere (Table 1). *Fagus grandifolia* is distributed widely in eastern North America (although the subsp. *mexicana* occurs in limited areas of Mexico) (Williams‐Linera et al. 2003), *Fagus sylvatica* is distributed broadly in central Europe, reaching locations in western Asia, and *Fagus crenata* is endemic and dominant in the Japanese archipelago, especially across the main islands (Matsui et al. 2004), corresponding to the latest divergence from western Eurasian *F. sylvatica* *s.l*. lineage in the Middle Eocene (Renner et al. 2016; Cardoni et al. 2022). The selected species show the closest morphological similarity, but differential evolutionary relatedness among them (Denk et al. 2024; Renner et al. 2016; Jiang et al. 2022; Denk and Grimm 2009).

The European species ( *F. sylvatica* ) adapts extraordinarily well to ecosystem variations and is established successfully under different understory conditions (Bradshaw et al. 2010; Packham et al. 2012; Muffler et al. 2021; Hara 2023). Despite all history related to early human settlements and their intervention in the distribution of the central European *Fagus* since Neolithic farming (Küster 1997; Bradshaw et al. 2010), for most of central Europe, the inherent natural vegetation consisted of *F. sylvatica* forming broad areas of monodominant beech forests with substantial morphological variation (i.e., taxonomically described as three subspecies, and 35 varieties within the species) (Hatziskakis et al. 2011). Stands of the other *Fagus* species selected from North America ( *F. grandifolia* ) and Asia ( *F. crenata* ) developed with relatively less human interference. Thus, both species can co‐occur with other species populations, forming mixed forests in some locations. In North America, *F. grandifolia* shares dominance with various species from deciduous genera (*Acer, Betula, Carya, Prunus, Quercus, Tilia, Liriodendron*) and rarely form monodominant patchy stands (Fang and Lechowicz 2006; Bradshaw et al. 2010). *Fagus grandifolia* is a major component of three of the Forest Inventory and Analysis (FIA) of forest cover types and a minor component in seventeen others. In Canada and the USA, the species is primarily distributed from the eastern Atlantic seaboard westward beyond the Appalachian Mountains into the Midwestern USA and Ontario, Canada, and from Nova Scotia and southern Quebec and southern Ontario, Canada, in the north, to northern Florida in the south, showing high morphological variations (i.e., three subspecies and six varieties). Regarding southern distributions, it reaches relicts of mixed montane forests in the northeast of Mexico (Galván‐Hernández et al. 2021). The Japanese beech (Siebold's beech), *F. crenata*, is endemic to Japan, where its distribution highlights distinct floristic elements in two main geographic regions. In eastern Japan, *F. crenata* forests are intermixed with other temperate tree species and frequently co‐dominate with other species of the genera *Torreya, Camellia* and *Ilex*. In western Japan, the species is also commonly found as a co‐dominant tree (Fang and Lechowicz 2006; Hara 2023). *Fagus crenata* distribution in Japan is limited by climate, which causes morphological differences among populations. Abiotic factors like light, temperature, precipitation, and snowfall vary across sites. These differences influence local horizontal and vertical distribution patterns and directly affect leaf structure (Hara 2010; Rooney et al. 2023; Ishizuka et al. 2012). The species is widely distributed along the Japanese archipelago, mainly in temperate and montane regions. Its primarily range covers the northeastern area of Honshu (the largest of the main islands), especially in the mountains of the Tohoku region. It extends south to the Takakuma Mountain in Kagoshima Prefecture on Kyushu Island. Its northernmost range reaches the southern and central mountain ranges of Hokkaido Island. Historical evidence indicates that during the Late Glacial period, approximately 12,000 years ago, Japanese beech expanded its range, concentrating mainly in areas with heavy snowfall south of 39°–40° N latitude. As the climate warmed, beech forest gradually retreated from the lowland of southwestern Japan, while simultaneously established themselves north of 40° N latitude, shaping pattern of distribution observed today (MAFF 2025).

### 2.2 Forest Inventory Data

The data compiled for the three species were extracted from published studies (search in Web of Science) and data repositories of the three regions (Table 2). Available public data for *F. grandifolia* was extracted from beech populations in the USA from the Fahey et al. (2022) dataset in the EDI Data Portal. We found extended information for *F. sylvatica* from several countries of Central Europe sourced from the ICP Forest repository. This data was initially compiled by ICP Forests Network, International Co‐operative Program on Assessment and Monitoring of Air Pollution Effects on Forests (ICP Forests 2022). Relevant data for *F. crenata* from Japan relied upon contributions from the Forestry and Forest Products Research Institute (FFPRI) in Japan (J. Nagakura, personal communication). Sample sizes of the three species are not balanced across sites due to limitations in data availability from public databases, publications and collaborator‐provided information. Data for *F. grandifolia* outside the USA are scarce or unavailable, while for *F. crenata*, information is fragmented, incomplete, and partly limited by language accessibility, with few studies focusing on specific foliar stoichiometry.

**TABLE 2:** The sample size for nutrient parameters and climate variables for the locations included in this study.

Parameter	*Fagus grandifolia* (*n*)	*Fagus sylvatica* (*n*)	*Fagus crenata* (*n*)
Nitrogen (N)	399	1610	17
Phosphorus (P)	399	1610	10
Potassium (K)	399	1610	11
Calcium (Ca)	396	1606	11
Magnesium (Mg)	397	1606	11
Sulfur (S)	0	1598	0
Sites	9	310	9
Elevation (m asl)	122–504	10–1748	6–134
MAP (mm)	1105 ± 10.7	930 ± 8.63	1524 ± 80.8
MAT (°C)	1.81 ± 0.73	8.53 ± 0.05	12.28 ± 0.39

### 2.3 Foliar Data Parameters and Climate

**FIGURE 1:**
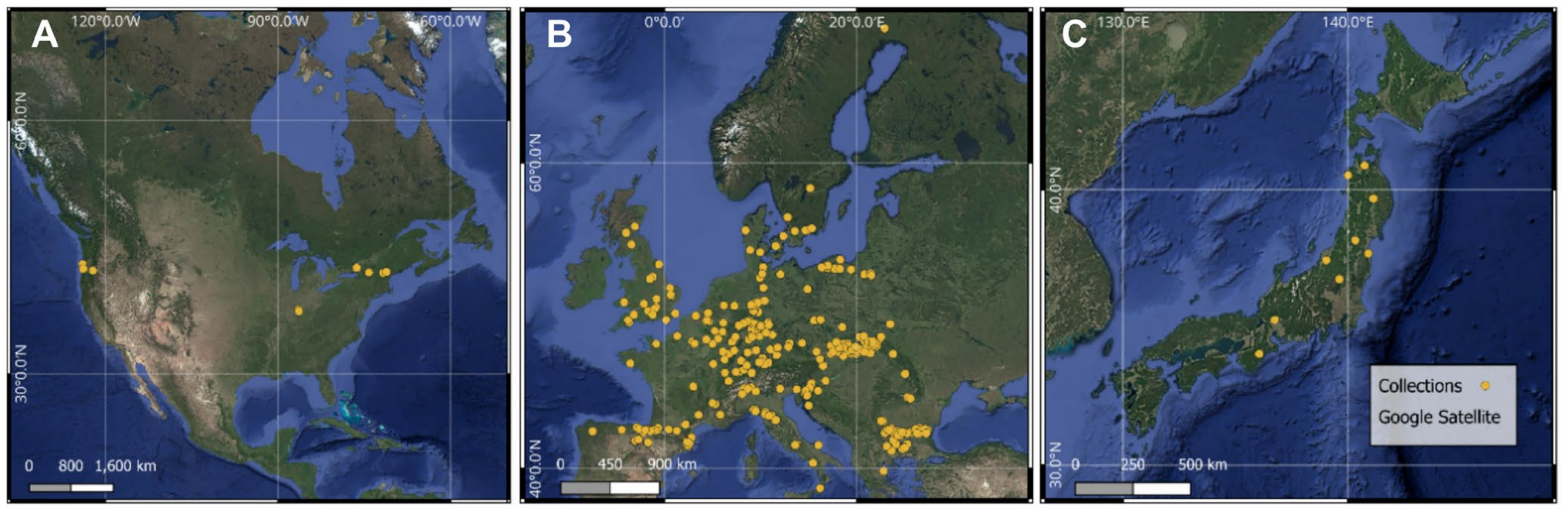
The foliar data used in this study were collected at study sites on three continents, and compiled in different data repositories: (A) in North America, *Fagus grandifolia* (*n* = 9) in east and west site of the USA; (B) in Europe, *Fagus sylvatica* (*n* = 310) in almost all countries of Central Europe; and (C) Asia, *Fagus crenata* (*n* = 9) along some populations in the main Honshu Island, Japan.

We used foliar concentrations of the nutrients N, P, K, Ca, and Mg as well as their ratios to characterize the biogeochemical niche of each of the three *Fagus* species: *F. grandifolia, F. sylvatica* and *F. crenata* in study sites from the USA, several EU countries, and Japan, respectively (Figure 1). Analogous to Sardans et al. (2015), we chose this broader set of elements, beyond N and P, because they are all very sensitive to plant functions and can better represent information about plant functions and the elementomes of our target species.

Data from all three databases were obtained using standardized and comparable methods for foliar nutrient content analyses across the 328 locations included in this study. Elemental analysis for *F. sylvatica* and *F. grandifolia* involved acid digestion. Nitrogen was determined primarily using the Kjeldahl method, while phosphorus was determined by colorimetric methods. Other macro‐ and micronutrients were analyzed from acid‐digested extracts using atomic absorption or emission spectroscopy, including AES, AAS, or equivalent techniques such as ICP‐AES or DCP‐AES (ICP Forests 2022; Fahey et al. 2022). For *F. crenata* , total nitrogen concentrations were determined using an NC analyzer (Sumigraph NC‐22F, Sumika Chemical Analysis Service, Osaka, Japan). Calcium, magnesium, potassium, and phosphorus concentrations were determined after wet digestion by HNO_3_ and HClO_4_ (2:1) using inductively coupled plasma optical spectrometry (ICP‐OES; Optima 4300DV, PerkinElmer, Norwalk, CT, USA) (J. Nagakura, personal communication). The main dataset was complemented by integrating main climate parameter data extracted from WorldClim. Using the geographical coordinates of the study sites, corresponding values for major bioclimate variables, such as mean annual temperature (MAT) from the BIO 1 layer and mean annual precipitation (MAP) from BIO 12, were extracted (Fick and Hijmans 2017). The primary data comprises samples collected between 1990 and 2016. Sample sizes used for this analysis are presented in Table 2.

### 2.4 Statistical Analysis

#### 2.4.1 Preliminary Bias Verification

We acknowledge that differences in sample size might introduce bias in our analyses. To ensure that the multivariate results were not driven solely by differences in sample sizes among regions, we conducted a sensitivity analysis using a randomly subsampled balanced dataset. The resulting Mahalanobis distances and species separations yielded consistent patterns, confirming the robustness of the biogeochemical niche differentiation observed in our results.

#### 2.4.2 Species Differentiation in BN

Species differentiation in foliar elemental composition and biogeochemical niche (BN) size was assessed using Functional Discriminant Analysis (FDA). Nutrient concentration (N, P, K, Ca, Mg) and nutrient ratios (N:P, N:K, N:Ca, N:Mg, P:K, P:Ca, P:Ma, K:Ca, K:Mg, Ca:Mg) were used as continuous predictor variables, whereas species were used as the grouping variable.

Discriminant analysis was performed to maximize species variation while minimizing within‐species variation, allowing the identification of species differentiation in the multivariate elemental space. Canonical scores resulting from the first and second discriminant roots were used to visualize species separation. Root 1 and Root 2 represent the canonical scores of the first and second discriminant functions, respectively.

To quantify the degree of separation among species in the multivariate elemental space, squared Mahalanobis distances (MD) were calculated between species centroids. These distances provide a measure of dissimilarity among species and serve as an indicator of niche differentiation in elemental composition. Statistical significance of species separation was conducted based on the discriminant analysis results. This analysis was performed in Statistical version 9 (StataCorp 2005).

#### 2.4.3 Influence of Climate and Foliar Nutrient Concentration

The influence of main climatic drivers on foliar composition was examined using mixed linear models (LMM) including mean annual precipitation (MAP) and mean annual temperature (MAT) as fixed effects, while species were treated as a random factor to account for non‐independence among observations. To account for spatial autocorrelation and unbalanced sampling, we used mixed‐effects models (LMM) with “Site” or “Region” as random factors where multiple trees were sampled per location, ensuring that species‐level imprints were not confounded by local edaphic hotspots. All models were implemented using lme function in nlme package (Pinheiro et al. 2024).

Finally, to account for correlation among observations within species and potential phylogenetic non‐independence, we conducted a comparative analysis using Generalized Estimation Equations (GEE) using the function Compar.gee in the ape library (Paradis and Schliep 2019). This method improves parameter estimation by explicitly accounting for clustered observations within species, which, in this case, are expected to be correlated. Accounting for this correlation improves the accuracy of the estimates. All statistical analyses were conducted in R (R Core Team 2025).

## 3 Results

**FIGURE 2:**
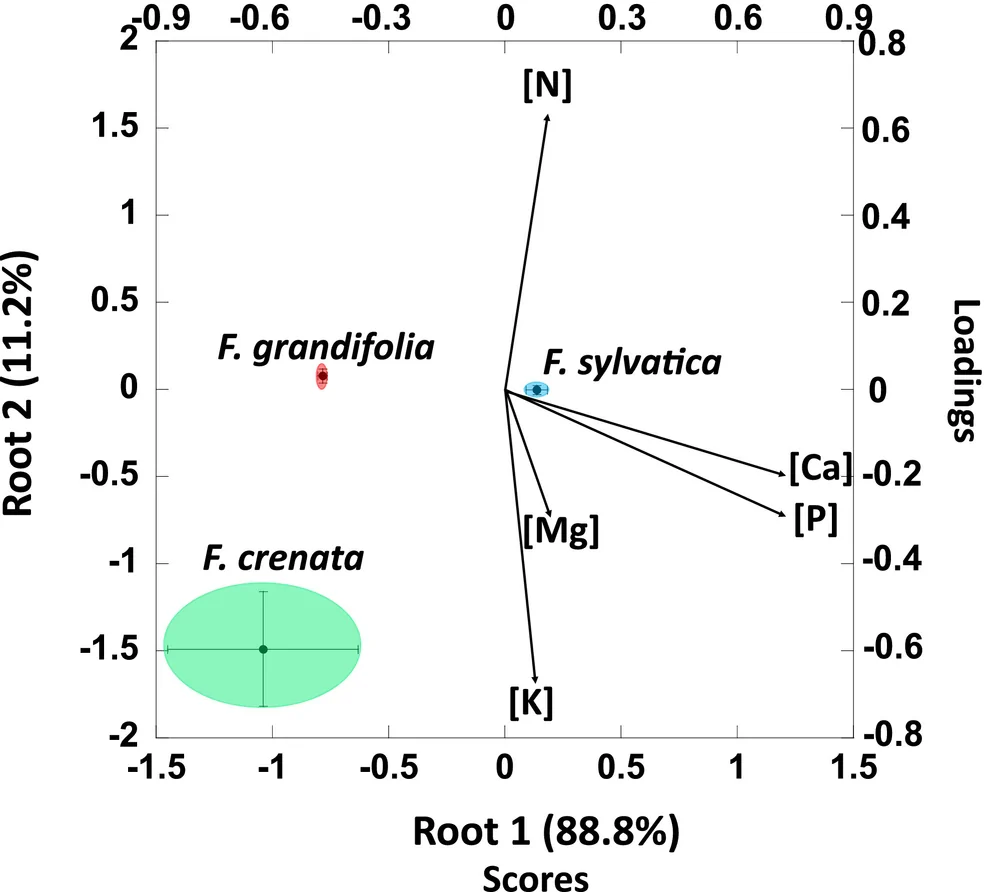
The representation of the 2‐D‐dimensional plot shows the separation of the three species. The colored surfaces represent the space occupied by at least 95% of the cases of each species.

Overall, our analysis confirmed the stoichiometrically species separation among *F. grandifolia*, *F. sylvatica* and *F. crenata* from the three study areas in the Northern Hemisphere (Figure 2). The five elements used to evaluate the elementome separation (i.e., N, P, K, Mg, and Ca) among the species showed significant evidence of functional divergence that validated our hypothesis (Tables 3 and 4).

**TABLE 3:** General discriminant analysis.

	Test	Value	*F*	Effect	Error	*p*
Intercept	Wilks	0.284	2369	2	1879	0.000000
N	Wilks	0.988	11.35	2	1879	0.000013
P	Wilks	0.950	49.46	2	1879	***p < 0.001***
K	Wilks	0.985	14.02	2	1879	0.000001
Mg	Wilks	0.994	5.80	2	1879	0.003077
Ca	Wilks	0.949	50.9	2	1879	***p < 0.001***

*Note:* Significance of each variable to the overall separation among/between groups. Bold italic values inidicate higly significant variables (*p* < 0.001).

**TABLE 4:** Comparative foliar nutrient concentration among the three *Fagus* species estimated using generalized estimating equations (GEE).

Variables	*Fagus grandifolia*	*Fagus sylvatica*	*Fagus crenata*	*Statistics*
N	2.37 ± 0.011a	2.40 ± 0.0102a	2.06 ± 0.11a	*F* = 5.86; *p = 0.0029*
P	0.112 ± 0.001**b**	0.135 ± 0.001**a**	0.122 ± 0.016**a**	*F* = 72.6; *P* < 0.0001
K	0.753 ± 0.007 ***c***	0.786 ± 0.006 ***b***	0.995 ± 0.105 ***a***	*F* = 9.03; *p* = 0.000125
Ca	0.615 ± 0.007**b**	0.883 ± 0.011**a**	0.712 ± 0.054**a**	*F* = 128.4; *P* < 0.0001
Mg	0.134 ± 0.002 ***b***	0.153 ± 0.002 ***a***	0.171 ± 0.013 ***a***	*F* = 12.6; *p* < 0.00001
N:P	21.5 ± 0.1**a**	19.2 ± 0.2**b**	18.9 ± 2.1**b**	*F* = 41.5; *P* < 0.00001
N:K	21.3 ± 0.2a	3.36 ± 0.04c	18.9 ± 2.1b	*F* = 11.097; *P* < 0.00001
N:Ca	4.13 ± 0.05a	3.40 ± 0.05b	3.13 ± 0.38b	*F* = 40.9; *p* < 0.00001
N:Mg	18.8 ± 0.2a	19.7 ± 0.3a	13.0 ± 1.5b	*F* = 4.38; *p* = 0.013
P:K	0.164 ± 0.003b	0.188 ± 0.003a	0.132 ± 0.020ab	*F* = 14.4; *p* < 0.00001
P:Ca	0.196 ± 0.003	0.190 ± 0.003	0.180 ± 0.028	*F* = 0.5829; *p* = 0.56
P:Mg	0.888 ± 0.010b	1.07 ± 0.02a	0.801 ± 0.166ab	*F* = 28.7; *p* < 0.00001
K:Ca	1.30 ± 0.02a	1.08 ± 0.02b	1.51 ± 0.25a	*F* = 34.6; *p* < 0.00001
K:Mg	5.97 ± 0.08b	6.27 ± 0.09a	6.13 ± 0.71ab	*F* = 2.38; *p* = 0.093
Ca:Mg	4.73 ± 0.05b	6.38 ± 0.07a	4.35 ± 0.37bc	*F* = 105; *p* < 0.00001
Root 1 canonical scores	0.654 ± 0,019	−0.305 ± 0.031	0.750 ± 0.306	*F* = 205; *p* < 0.00001
Root 2 canonical scores	0.02775 ± 0.029	−0.0016 ± 0.0294	−1.617 ± 0.40	*F* = 13.3; *p* < 0.00001

*Note:* Group differentiations are indicated by lowercase letters. The significance of the explanatory variables (nutrient elements) in the models is shown by ** (*p* < 0.01) and *** (*p*‐value < 0.001).

### 3.1 Species Functionality Variation by Climate

Analyses using linear mixed (LMMs) that included MAT and MAP as primary climatic variables indicated that most of the statistically significant variation was attributable to the random component of species identity, as determined by model comparison testing (Table 5). These results suggest that species‐specific legacy effects may account for much of the variance in leaf elemental composition. Foliar P was primarily explained by species identity, showing the strongest species effect among the elements analyzed. In contrast, Ca and K showed moderate species effects, with additional minor contributions from climate variables. The variance among species appears to be marginally influenced by environmental differences experienced during their life histories and may reflect genetically encoded differences (Table 5).

**TABLE 5:** Linear mixed model with mean annual precipitation (MAP) and mean annual temperature (MAT) as fixed factors and species as random factor effects on stoichiometrical variables.

Variable	Variable ~ map*mat, data = dades, random = ~1 | species, method = ‘ML’, na.action = na.omit							
	Fixed factors					Random effects		Statistics
		numDF	denDF	*F*	*p*	Formula: ~1 | species		
N	(Intercept)	1	2024	563.5	< 0.0001	(Intercept)	Residual	*R* ^2^m = 0.011
	map	1	2024	2.17	0.14	StdDev: 0.1582557	0.3434394	***R*** ^**2**^ **r = 0.165**
	mat	1	2024	16.3	0.0001			*R* ^2^c = 0.18
	map:mat	1	2024	0.717	0.40			
P	(Intercept)	1	2024	296.0	< 0.0001	(Intercept)	Residual	*R* ^2^m = 0.056
	map	1	2024	87.3	< 0.0001	StdDev: 0.01114691	0.03905	***R*** ^**2**^ **r = 0.74**
	mat	1	2024	10.1	0.0015			*R* ^2^c = 0.13
	map:mat	1	2024	30.6	< 0.0001			
K	(Intercept)	1	2024	102.0	< 0.0001	(Intercept)	Residual	*R* ^2^m = 0.020
	map	1	2024	27.5	< 0.0001	StdDev: 0.1376208	0.22292	***R*** ^**2**^ **r = 0.27**
	mat	1	2024	0.168	0.68			*R* ^2^c = 0.29
	map:mat	1	2024	13.74	0.0002			
Ca	(Intercept)	1	2024	94.6	< 0.0001	(Intercept)	Residual	*R* ^2^m = 0.0042
	map	1	2024	6.05	0.014	StdDev: 0.1211482	0.3464191	***R*** ^**2**^ **r = 0.1098**
	mat	1	2024	0.606	0.44			*R* ^2^c = 0.113
	map:mat	1	2024	2.81	0.094			
Mg	(Intercept)	1	2024	503.9	< 0.0001	(Intercept)	Residual	*R* ^2^m = 0.00079
	map	1	2024	0.982	0.3218	StdDev: 0.0090857	0.076610	***R*** ^**2**^ **r = 0.01381**
	mat	1	2024	0.094	0.7587			*R* ^2^c = 0.0146
	map:mat	1	2024	0.546	0.4600			
N/P	(Intercept)	1	2024	400.0	< 0.0001	(Intercept)	Residual	*R* ^2^m = 0.059
	map	1	2024	90.0	< 0.0001	StdDev: 1.576507	4.98237	***R*** ^**2**^ **r = 0.096**
	mat	1	2024	17.1	< 0.0001			*R* ^2^c = 0.155
	map:mat	1	2024	30.8	< 0.0001			
N/K	(Intercept)	1	2024	10.62	0.0011	(Intercept)	Residual	*R* ^2^m = 0.00128
	map	1	2024	8.12	0.0044	StdDev: 7.681853	2.520	***R*** ^**2**^ **r = 0.8902**
	mat	1	2024	2.27	0.13			*R* ^2^c = 0.903
	map:mat	1	2024	6.20	0.013			
N/Ca	(Intercept)	1	2024	318.5	< 0.0001	(Intercept)	Residual	*R* ^2^m = 0.0078
	map	1	2024	2.23	0.14	StdDev: 0.3047725	1.67985	***R*** ^**2**^ **r = 0.0312**
	mat	1	2024	3.07	0.08			*R* ^2^c = 0.039
	map:mat	1	2024	4.87	0.027			
N/Mg	(Intercept)	1	2024	3927.9	< 0.0001	(Intercept)	Residual	*R* ^2^m = 0.00087
	map	1	2024	1.05	0.31	StdDev: 0.3040762	9.64535	***R*** ^**2**^ **r = 0.00083**
	mat	1	2024	0.180	0.67			*R* ^2^c = 0.0017
	map:mat	1	2024	0.540	0.46			
P/K	(Intercept)	1	2024	168.2	< 0.0001	(Intercept)	Residual	*R* ^2^m = 0.0405
	map	1	2024	12.8	0.0004	StdDev: 0.01964502	0.097292	***R*** ^**2**^ **r = 0.7395**
	mat	1	2024	3.00	0.084			*R* ^2^c = 0.078
	map:mat	1	2024	64.2	< 0.0001			
P/Ca	(Intercept)	1	2024	6050.2	< 0.0001	(Intercept)	Residual	*R* ^2^m = 0.011
	map	1	2024	1.06	0.30	StdDev: 2.532471e−06	0.11	*R* ^2^r = 0.006
	mat	1	2024	0.986	0.32			*R* ^2^c = 0.017
	map:mat	1	2024	19.9	< 0.0001			
P/Mg	(Intercept)	1	2024	234.8	< 0.0001	(Intercept)	Residual	*R* ^2^m = 0.021
	map	1	2024	22.6	< 0.0001	StdDev: 0.0923694	0.5241113	***R*** ^**2**^ **r = 0.029**
	mat	1	2024	3.27	0.071			*R* ^2^c = 0.050
	map:mat	1	2024	17.86	< 0.0001			
K/Ca	(Intercept)	1	2024	197.8	< 0.0001	(Intercept)	Residual	*R* ^2^m = 0.0031
	map	1	2024	0.241	0.62	StdDev: 0.1335639	0.5690	***R*** ^**2**^ **r = 0.0519**
	mat	1	2024	1.630	0.20			*R* ^2^c = 0.055
	map:mat	1	2024	4.497	0.03			
K/Mg	(Intercept)	1	2024	8994.5	< 0.0001	(Intercept)	Residual	*R* ^2^m = 0.0056
	map	1	2024	2.35	0.13	StdDev: 8.280173e−05	2.92993	*R* ^2^r = 0.0002
	mat	1	2024	0.464	0.50			*R* ^2^c = 0.0058
	map:mat	1	2024	8.51	0.0036			
Ca/Mg	(Intercept)	1	2024	90.0	< 0.0001	(Intercept)	Residual	*R* ^2^m = 0.0013
	map	1	2024	2.68	0.10	StdDev: 0.8900813	2.406499	***R*** ^**2**^ **r = 0.1197**
	mat	1	2024	0.032	0.86			*R* ^2^c = 0.121
	map:mat	1	2024	0.106	0.75			

*Note:* *R* ^2^m = percentage of the variance explained by fixed factors. *R* ^2^r = percentage of variance explained by random (species). *R* ^2^c = percentage of variance explained by the overall model. Bold *R* ^2^r values indicate cases where the variance attributed to the random effect (species) is statistically significant based on model comparison testing (*p* > 0.05).

### 3.2 Elementome Relationships Among Species

Each nutrient element contributed differently to distinguishing among species, as shown by their varying statistical significance (Tables 3 and 6). All five elements demonstrated statistically significant contributions (*p*‐value < 0.005), indicating that each element helps differentiate the three species within the multivariate elemental space.

**TABLE 6:** Level of separation in the *n*‐dimensional space generated for the five elements concentration used and continuous independent analyses in the discriminant analysis.

	*Fagus sylvatica*	*Fagus crenata*
*Fagus grandifolia*	MD = 0.857 *F* = 39.4 *p < 0.0001*	MD = 2.52 *F* = 5.31 *p < 0.0001*
*Fagus sylvatica*		MD = 3.60 *F* = 7.84 *p < 0.0001*

*Note:* Squared Mahalanobis distances (MD) are the statistic that is a proxy of Euclidean distances among the cases (individual data) mean ± confidence interval (95%) among different groups of cases (in this case, *Fagus* spp.). *F*‐Fisher statistics is the equivalent value of MD in these other statistics, and finally, we show the corresponding *p*‐value.

The GEE analyses revealed, to a greater extent, the significant differences among the *Fagus* species for all nutrient elements and ratios evaluated (Table 4). The three species have comparable N content. The content of P and Ca differed among species. Hence, P and Ca are the elements that separate *F. grandifolia* from the other two species. In contrast, K concentrations differ among the three species (Table 4). Specifically, *F. grandifolia* exhibited the highest N:P ratio and generally lower concentrations of P, K, Ca and Mg. In comparison, *F. sylvatica* showed the highest values of P and Ca. Furthermore, *F. crenata* was distinguished by the highest K and Mg concentration, while its N:P ratio was the lowest among the three species.

Additionally, the functional discriminant analysis (FDA) of foliar elemental composition (N, P, K, Ca, and Mg) confirmed all five elements contributed significantly to species separation (Wilks' Lambda = 0.284, *p* < 0.0001), with the first canonical root explaining 88.8% of the total variance (Tables 3 and 6). In the two‐dimensional discriminant space defined by the canonical roots (Figure 2), the squared Mahalanobis distances (MD) indicate a clear significant functional separation among the three species (Table 6). The greatest distance was observed between *F. sylvatica* and *F. crenata* (MD = 3.60). This was followed by *F. grandifolia* and *F. crenata* (MD = 2.52), while *F. grandifolia* and *F. sylvatica* were moderately separated (MD = 0.86). These results show that the foliar elemental profiles of the species are distinct in a multivariate space (Figure 2). *Fagus sylvatica* and *F. crenata* show the most contrasting overall elemental composition. The wider dispersion observed for MD in *F. crenata* reflects also the differences in sample size relative to other species. Nevertheless, *F. crenata* occupied a clear distinct position in the multivariate space, separated from the other two species. Consistent with this limitation in the analysis, supplementary analyses supported the same pattern (Appendix S1, Table S1).

## 4 Discussion

In this study, we found that the three species have distinct, individual biogeochemical niches, with clear separation in the multivariate space defined by elemental composition (N, P, K, Mg, Ca). The results also confirm that variation in unique elemental composition (elemetome) is determined primarily by species identity (Table 4). This indicates that each species possesses its own mechanisms for regulating nutrient allocation and use, an intrinsic feature of its functional strategy. The elemental composition of each species provides information on its ecological strategies that characterize their performance and likely contribute to their ecological success across the three study regions.

Consequently, our results contribute to a better understanding of the biogeochemical processes that determine patterns of growth, respiration and survival in each species. Moreover, interpreting elementome variation within established conceptual frameworks, such as Leaf Economics Spectrum (Wright et al. 2004) and strategy theory (Grime 1977; Grime et al. 1997; Aerts and Chapin III 1999), enables the identification of traits that reflect more acquisitive (fast return) or more conservative (resources retaining) functional strategies among the species.

While these patterns highlight strong taxonomic structuring, we acknowledge that historical and biogeographical processes have also influenced the current ecological dominance of some species. For instance, the extensive dominance of *F. sylvatica* in Central Europe has been associated with long‐term human modification of forest landscapes (Bradshaw et al. 2010). Although these historical processes are unlikely to determine the species intrinsic functional strategies, they may promote the ecological success and widespread expression of those strategies under present day conditions. Importantly, as anthropogenic climate modifications alter forest composition, characterizing the biogeochemical niches of these *Fagus* species becomes increasingly significant because it enables us to anticipate how species will respond to future changes in temperate ecosystems.

### 4.1 Biogeochemical Niches and Functional Strategies

Elementome analysis shown differentiated BN among the three species, suggesting variation in their competitive abilities and ecological roles. This separation is also clear in the multidimensional space from discriminant analysis and MD distance values (Table 6). The differentiation of elementomes among the three species is characterized by combinations of nutrient ratios that separate them in multidimensional space and reflect functional strategies that differentiate them along an acquisitive–conservative spectrum (Reich et al. 1997; Wright et al. 2004).

We found that *F. sylvatica* notably exhibits higher concentrations of P and Ca, with intermediate N:P values relative to the other species, traits characteristic of acquisitive species. High concentrations of nutrients essential for growth and a low N:P ratio indicate active metabolism and a high capacity for resource acquisition (Wright et al. 2004; Diaz et al. 2004). This high metabolic investment likely facilitated its rapid post‐glacial colonization across Europe. Furthermore, the elevated foliar Ca concentrations align with its competitive advantage on calcareous substrates, suggesting that *F. sylvatica* has evolved to maximize nutrient throughput to maintain dominance across diverse soil environments. Human management has influenced its current distribution, but the acquisitive nature of its elementome is a pre‐existing genetic trait that likely enabled its success under human‐altered disturbance regimes. In Germany, Leuschner et al. (2006) reported a broad range of soil types occupied by beech trees with some stands showing a physiological advantage on calcareous (lime‐rich) substrate, associated with drought stress resilience. *Fagus sylvatica* prefers warmer sites and is also dominant on calcareous soils (Leuschner et al. 2006), consistent with the high concentration of foliar Ca among the species in this study. However, European beech can be found as well in acidic soils (Cambisol and Luvisols) in regions of northern Germany, France and the UK, where it cohabits dominantly with few other species of hardwood trees (Holzwarth et al. 2011). Its presence on various types of soil throughout Europe depict the acquisitive features this species showed in our results. We hypothesized that *F. sylvatica* would exhibit elevated P concentration and low N:P ratios reflecting traits associated with rapid growth. This hypothesis was only partially supported by the data. Previous evidence showed that *F. sylvatica* can adjust foliar P concentration in response to soil P availability (Meller et al. 2019). In our data, *F. sylvatica* showed relatively high foliar concentration of P and Ca compared to other species, while foliar N concentration did not differ significantly among species. European beech appears to show a comparatively higher degree of responsiveness to environmental gradients such as soil pH and nutrient availability that its North American and Asian congeners.

At the other extreme, *F. grandifolia* presents the lowest values of P, K, Ca, and Mg, and the highest N:P values. This pattern is consistent with a conservative strategy, characterized by efficient resource use, lower growth rate, and greater nutrient conservation (Aerts and Chapin III 1999; Wright et al. 2004; Reich and Oleksyn 2004). This allows the species to achieve a competitive equilibrium with co‐occurring deciduous trees in the highly diverse mixed forests of North America. While soil parent material undoubtedly influences foliar chemistry, the persistence of these species‐specific signatures across vast geographic distances suggests that genetic homeostasis effectively overrides local edaphic variation in the genus *Fagus*. Thus, our findings reveal traits compatible with persistence and stability in species communities. *F. grandifolia* achieves competitive equilibrium with co‐occurring species. Ecological studies based on the current distribution ranges and dominance or co‐dominance assemblages of this species suggest the features observed in this study. *F. grandifolia* is efficient in its adaptive success, such as highly shade‐tolerance, long term survival under low light (Gravel et al. 2008). It is not a dominant species but serves as a keystone species in mid‐temperate forests near both the north and south distribution limits (Cogbill 2005; Harcombe et al. 2002). The species diversified and adapted from north to south in eastern North America, forming mixed forests where it is dominant or co‐dominant. Interestingly, based on an analysis of litter leachate chemistry from *F. grandifolia* from Maine to North Carolina, Hudson et al. (2018) found P concentrations of litter leachate were the most influential factor delineating between geographically (and genetically) separated populations of *F. grandifolia* . The data available for this study mainly cover sites in the northeast and northwest of the species' range (Figure 1), where its leaf chemistry has been studied in more detail. However, taxonomic and ecological studies in the US range indicate that the species is a mature forest species, highly tolerant of shade. From the existing literature, we found that throughout its range, *F. grandifolia* forms at least seven forest types (Greller et al. 2018), which include floristic elements from the Boreal, deciduous, and subtropical/tropical zones. This leads to functional variation in the species. Further southern populations are less studied. For instance, populations' distributions in Mexico are completely isolated from the distribution continuum though the east site of North America and show other unique assemblages such as patches of monodominance together with a high genetic differentiation due to reduced exchange with the populations in the USA (Galván‐Hernández et al. 2021; Williams‐Linera et al. 2003). Thus, from the Mexican populations of *F. grandifolia*, we expect more differentiated stoichiometric variation of the species strategy.

For *F. crenata*, elevated concentrations of K and Mg, along with intermediate levels of P and Ca and the lowest N:P ratio, indicate a more acquisitive nutrient strategy. Although the difference in N:P compared to *F. sylvatica* is not statistically significant (Table 4), these findings point to a high capacity for nutrient acquisition and utilization. *F. crenata* therefore occupies an intermediate acquisitive—conservative position among the studied species. The substantial variability observed in K and N:P ratios may reflect greater physiological plasticity in response to environmental conditions, rather than a strictly conservative strategy. This plasticity likely represents an adaptation to the high‐snowfall, nutrient‐leached environments of the Japanese archipelago, where nutrient retention is more critical for survival than rapid metabolic growth. Ecological studies also indicate moderate acquisitive capacity when resource availability improves. For example, Japanese beech demonstrates increased growth under higher light availability, and other species that has a stable growth under shade (Shimano 2002; Yoshida and Kamitani 1998). Conversely, *F. crenata* exhibits conservative traits such as high shade tolerance, stable growth, and dominance in mature forests (Osada et al. 2004). Additional ecological studies comparing Japanese beech associations with diverse floristic communities and geographic distributions have established that climatic conditions are closely linked to its life history (Momohara and Ito 2023; Hara 2023). The influence of climate is evident in the biogeographical distribution of *F. crenata*, which displays distinct characteristics in species assemblages and intraspecific variability (Hara 2010, 2023). Our results capture a portion of the physiological plasticity exhibited by this species. Thus, it would be premature to assign *F. crenata* to a single functional category based on its elementome without more comprehensive data across its full distribution range in Japan.

### 4.2 Implications and Future Research

This study provides an initial but robust assessment of the functional strategies of ecologically important taxa in temperate forest ecosystems. The evolutionary history of these species differs substantially, and their interactions with human‐driven environmental change remain an important topic for further investigation. Although our dataset captures meaningful variation among the three studied species, differences in data availability across regions may have influenced the resolution of trait–environment relationships, and in the classification from homeostatic versus plastic and from acquisitive to conservative strategies. In addition, the lack of detailed soil information for all study sites represents an important limitation in fully resolving the drivers of stoichiometric variation. To better understand the role of phylogenetic constraints in shaping species‐specific biogeochemical niches, future studies should incorporate explicit phylogenetic frameworks together with a broader sampling of taxa within the Fagaceae family. Expanding both the number of species and the environmental context will be essential to fully characterize functional diversity within this family and to clarify the mechanisms underlying their ecological and evolutionary success in temperate forest ecosystems.

## 5 Conclusion

In summary, our study reveals significant biogeochemical niche divergence across the global distribution of the genus *Fagus*. The contrasting strategies from the acquisitive *F. sylvatica* to the conservative *F. grandifolia* are consistent with an evolutionary trade‐off between growth potential and environmental resilience, while *F. crenata* exhibits the strongest climatic modulation of its elementome among the three beech species, suggesting greater functional flexibility in nutrient allocation across climatic gradients. These species‐specific nutrient strategies may provide a mechanistic basis for predicting forest transitions and competitive outcomes under future climate scenarios. Our study demonstrates that the “Darwinian shortfall” in forest biogeochemistry can be addressed by integrating elementome analysis into evolutionary frameworks. By linking phylogenetic history, elemental composition, and functional strategies, our results provide a promising basis for predicting the functional restructuring of Northern Hemisphere forests under future anthropogenic change.

## Author Contributions

**Daisy Cárate Tandalla:** data curation (equal), visualization (equal), writing – original draft (lead), writing – review and editing (lead). **Hermann F. Jungkunst:** conceptualization (equal), investigation (equal), supervision (supporting), writing – review and editing (supporting). **Janice E. Hudson:** data curation (equal), investigation (lead), resources (equal), writing – review and editing (supporting). **Delphis F. Levia:** investigation (supporting), supervision (equal), writing – review and editing (equal). **Junko Nagakura:** investigation (supporting), resources (equal), writing – review and editing (supporting). **Josep Peñuelas:** conceptualization (lead), formal analysis (equal), investigation (equal), methodology (equal), supervision (supporting), writing – review and editing (equal). **Vanessa M. S. Vetter:** conceptualization (lead), investigation (equal). **Jordi Sardans:** conceptualization (equal), data curation (lead), formal analysis (lead), methodology (lead), visualization (equal), writing – review and editing (lead).

## Funding

The authors have nothing to report.

## Conflicts of Interest

The authors declare no conflicts of interest.

## Supporting information


**Table S1:** Squared Mahalanobis distances among the three centroids of different *Fagus* species in function of their distribution foliar N, P, K, Ca and Mg concentrations. The statistical significance between pairwise differences among species is also indicated.

## Data Availability

The dataset supporting this study has been deposited in the Dryad Digital Repository (DOI: https://doi.org/10.5061/dryad.qfttdz0wb).
