# Supplementary material for: Evolutionary Imprints on the Foliar Elementome: Biogeochemical Niche Differentiation Among Fagus Species
Source: Ecol Evol. 2026 Aug 26;16(9):e74196. doi: 10.1002/ece3.74196 (PMC13519295; doi:10.1002/ece3.74196)
Supplement: Supplementary file 1 [file ECE3-16-e74196-s001.docx]

**Evolutionary Imprints on the Foliar Elementome: Biogeochemical Niche Differentiation Among *Fagus* Species**

**Supporting information**

**Table S1.** Squared Mahalanobis distances among the three centroids of different *Fagus* species in function of their distribution foliar N, P, K, Ca and Mg concentrations. The statistical significance between pairwise differences among species is also indicated.

|  | ***Fagus grandifolia*** | ***Fagus crenata*** |
| --- | --- | --- |
| ***Fagus sylvatica*** | M = 0.94  F = 81.4  **P <0.0001** | M = 3.73  F = 7.38  **P<0.0001** |
| ***Fagus grandifolia*** |  | M = 2.71  F = 5.33  **P<0.0001** |
